# mRNA vaccines for COVID-19: what, why and how

**DOI:** 10.7150/ijbs.59233

**Published:** 2021-04-10

**Authors:** Jung Woo Park, Philip N.P. Lagniton, Yu Liu, Ren-He Xu

**Affiliations:** Institute of Translational Medicine, and Centre of Reproduction, Development and Aging, Faculty of Health Sciences, University of Macau, Taipa, Macau, China.

**Keywords:** COVID-19, SARS-CoV-2, mRNA vaccine, efficacy and safety

## Abstract

The Coronavirus disease-19 (COVID-19) pandemic, caused by severe acute respiratory syndrome coronavirus -2 (SARS-CoV-2), has impacted human lives in the most profound ways with millions of infections and deaths. Scientists and pharmaceutical companies have been in race to produce vaccines against SARS-CoV-2. Vaccine generation usually demands years of developing and testing for efficacy and safety. However, it only took less than one year to generate two mRNA vaccines from their development to deployment. The rapid production time, cost-effectiveness, versatility in vaccine design, and clinically proven ability to induce cellular and humoral immune response have crowned mRNA vaccines with spotlights as most promising vaccine candidates in the fight against the pandemic. In this review, we discuss the general principles of mRNA vaccine design and working mechanisms of the vaccines, and provide an up-to-date summary of pre-clinical and clinical trials on seven anti-COVID-19 mRNA candidate vaccines, with the focus on the two mRNA vaccines already licensed for vaccination. In addition, we highlight the key strategies in designing mRNA vaccines to maximize the expression of immunogens and avoid intrinsic innate immune response. We also provide some perspective for future vaccine development against COVID-19 and other pathogens.

## Brief introduction of the coronavirus disease 2019 (COVID-19)

The COVID-19 pandemic has thus far caused infection of more than 100 million people and over 2 million deaths worldwide. These numbers also reflect an astonishing increase compared to more than 80,000 infections and over 3,000 deaths by March 2020 1 when the World Health Organization declared COVID-19 a world pandemic. Moreover, the pandemic brought nearly the entire world to stop due to the consequent global crisis in health, economy, and psychology. COVID-19 is caused by severe acute respiratory syndrome coronavirus -2 (SARS-CoV-2), a name adapted from SARS-CoV that caused the infection of SARS in 2003 2. Since none of the explored therapies can directly kill the virus, vaccines have become the last hope to stop the pandemic. However, vaccine development is generally a time-consuming process, taking years to complete. As a great surprise, Moderna Biotechnology, Inc. delivered a vaccine named mRNA-1273 in only 42 days from the date when the spike protein-coding sequence of SARS-CoV-2 was published on January 10, 2020 3. Together, it took less than one year to complete the design, manufacture, efficacy and safety tests, and evaluation and approval for use.

Both mRNA vaccines have been found to be highly effective and safe in preventing COVID-19 according to clinical trials 4, 5. Compared to other vaccine platforms, mRNA vaccines possess unique advantages including versatility, efficient delivery, use of the protein translational machinery of the host, and short developmental time. In this review, we introduce the general principles for the design, optimization, working mechanisms, and challenges of mRNA vaccine development. In addition, we will summarize up-to-date clinical trial data on mRNA vaccines. Hopefully, the review will help readers comprehend the history, current status, and prospective of mRNA vaccines for immunization against COVID-19 and also future infectious diseases.

### Etiology and pathophysiology of COVID-19

COVID-19 is caused by SARS-CoV-2, which is an enveloped virus with a positive-strand and single-stranded RNA genome that belongs to the β-coronavirus subfamily 6. The RNA genome of SARS-CoV-2, approximately 30 kb, encodes 14 open reading frames. The 5'-proximal end of the genome encodes two polypeptides, pp1a and pp1b, by a programmed -1 ribosomal frameshift 7. Two polypeptides are cleaved into 16 non-structural proteins (nsp1-16), which mediate the delivery of the viral replication complexes to subcellular domains and viral replication, transcription, and post-transcriptional processes 8. A surface envelope glycoprotein, Spike (S), encoded by the 3,822-bp S gene, mediates the viral entry into host cells via binding to its functional receptor, angiotensin-converting enzyme 2 (ACE2), expressed highly in lung alveolar epithelial cells and epithelial cells of many other organs including the heart, kidney, bladder, and ileum 9, 10. Therefore, these organs are considered more vulnerable to SARS-CoV-2 11. The primary symptoms of COVID-19 include fever, dry cough, shortness of breath, muscle ache, dizziness, headache, sore throat, rhinorrhea, chest pain, diarrhea, nausea, and vomiting 1. When the viral load is high or when the infection happens in patients with other severe diseases, the patients often develop acute respiratory syndrome and sepsis in a short time 1.

### Mutation rate and transmissibility of SARS-CoV-2

RNA viruses such as influenza virus and HIV typically undergo mutation at a much higher rate than DNA viruses because RNA viruses usually lack the proof-reading activity. Although SARS-CoV-2 possesses the proof-reading activity, facilitated by nsp14 (Exonuclease N)-nsp10 complexes 12, scientists cataloged more than 12,000 different mutations in the SARS-CoV-2 genome 13. Recently, several mutant variants including D614G mutation have replaced the original Wuhan strain and spread as dominant strains, possibly due to their increased infectivity to ACE2 14, 15. The South African strain, known as 501.V2 or B.1.351, underwent three amino acids substitutions, K417N, E484K, and N501Y in S protein of the original strain, among which N501Y is located in the receptor-binding domain (RBD).

Another variant recently identified in the UK, known as B1.1.7 or VOC202012/01, contains eight mutations in S protein, among which the N501Y mutation is overlapped with 501.V2 variant. Although the overall effect of the mutations is not yet known, the high transmission rate of UK iB1.1.7 in the UK may result from the increased affinity of the N501Y mutation to ACE2. In fact, a recent computational analysis predicts that the N501Y mutation likely results in an increased number of interactions with the Y41 and K353 amino acids of ACE2 hence elevated affinity 16. There is a growing concern that the current vaccines may not protect people from the newly emerging variants of SARS-CoV-2. However, the faster development of mRNA vaccines than the other vaccine types may be a solution to prevent against the current and future variants as well as other outbreaks of viral diseases like COVID-19.

## Vaccine development for previous coronavirus epidemics

During the outbreaks of Severe Acute Respiratory Syndrome (SARS) in 2002-2004 and Middle East Respiratory Syndrome (MERS) in 2012, vaccines were developed for these diseases but never licensed for humans. Two vaccine types, one inactivated form of SARS-CoV-1 virus developed by Sinovac Biotech Ltd. and one DNA-based vaccine developed by National Institute of Allergy and Infectious Disease (NIAID), were tested for phase I clinical trials but never licensed for human vaccination and commercial use 17, 18. The majority of the vaccine development against SARS-CoV-1 was completely dropped since the virus never re-emerged after the first outbreak. As for MERS coronavirus, nine vaccines derived from various platforms were tested for phase I/II clinical trials 19, 20. For example, ChAdOx developed by the University of Oxford was based on the replication-deficient adenovirus vector, expressing full-length S protein of MERS-CoV 21. The coding sequence was optimized for protein translation. As demonstrated in animal studies, the vaccine was highly immunogenic and able to activate CD8+ T cells for exerting cytotoxicity and B cells for producing neutralizing antibodies 21. It was also documented that two of 42 anti-MERS mRNA vaccine candidates were developed and tested at the pre-clinical stage, but neither proceeded to a large-scale clinical trial to be a licensed vaccine 22. Currently, 63 anti-SARS-CoV-2 candidate vaccines have been being tested on clinical trials (Table 1). Although mRNA vaccines represent only 11% of all the vaccines developed on various platforms, two mRNA vaccines, mRNA-1273 (developed by Moderna) and BNT162b (developed by Pfizer and BioNTech Ltd.) were the first vaccines approved for emergency use in many countries.

### History of mRNA vaccines

mRNA vaccine is based on the principle that mRNA is an intermediate messenger to be translated to an antigen after the delivery into host cells via various routes. RNA molecules have been utilized as therapeutic and research tools for more than two decades, with the usage ranging from *in vitro* transcribed (IVT) mRNA, small interference RNA (siRNA), RNA aptamers, riboswitches, antisense RNA to the recent developed mRNA vaccines 23-26. The idea that mRNA molecules can be directly delivered into cells for manipulating gene expression or producing proteins of interest was first tested in the late 1980s. Malone, *et al.*, first demonstrated the efficient transfection of mRNA in NIH 3T3 fibroblasts using a cationic lipid N-[1-(2,3-dioleyloxy)propyl]-N,N,N-trimethylammonium chloride 27. The idea of transfecting mRNA molecules into host cells for the expression of a gene of interest underwent several technical improvements over the next two decades or so 28.

In early 1990s, direct expression of external mRNA molecules in host animals for therapeutic effects was first tested by delivering RNA vectors encoding a reporter gene such as luciferase and β-galactosidase into murine muscle cells and transfecting vasopressin mRNA into rats to reverse Diabetes-Insipidus 29, 30. In 1993 Martinson, *et al.*, demonstrated that an *in vitro* synthesized mRNA vaccine encoding nucleoprotein of influenza virus triggered the activation of cytotoxic T lymphocytes in mice 31. Later, it was found that *in vivo* application of mRNA induced both activation of cytotoxic T cells and humoral response of B cells to produce specific antibodies 32.

However, the possibility of using mRNA as a vaccine was not seriously taken due to the easy degradability of RNA, the ubiquitous presence of ribonucleases, and the lack of scalability. A series of advancements occurred recently when mRNA vaccines were used to prevent triple-negative breast cancer and lung carcinoma using mRNAs encoding MUC1 and herpes simplex virus I thymidine kinase, respectively 33, 34. The application has been utilized in prevention of cancer and infectious diseases and treatment of allergy and other diseases that need protein replacement. Numerous mRNA vaccines are under clinical trials or already available today against infectious pathogens such as Zika virus, cytomegalovirus, influenza virus, metapneumovirus, and parainfluenza virus as well as cancer 35.

### Advantages and disadvantages of mRNA vaccines over other vaccine platforms

The vaccine development can generally be classified into two categories: gene-based and protein-based. The protein-based approach has been the conventional method that relies on attenuated or recombinant proteins directly delivered as immunogens to activate the adaptive and humoral immune response. The gene-based vaccines are delivered via a DNA or RNA vector to host cells where they will be expressed to produce corresponding antigens to induce the immune response in the host. Both protein- and gene-based vaccines (including DNA and RNA) have been explored for COVID-19 and currently on clinical trials. There are several advantages of mRNA vaccines over the other platforms.

The first advantage of mRNA vaccines is the easiness and fast speed for their manufacturing. The core principle of mRNA vaccines is to deliver a transcript that encodes a target antigen or immunogen. The RNA synthesis can immediately be carried out on the same platform as soon as the sequence encoding the immunogen is available and the process can be easily scalable and cell-free, requiring minimal platform change during mRNA formulation and manufacturing 36. Second, a mRNA vaccine expresses target protein (antigen) via translation from the mRNA rapidly after its transfection. mRNA vaccines possess much higher biosafety than DNA-based vaccines as the translation of the antigens takes place in the cytoplasm rather than the nucleus, thus much less possible for the mRNA to integrate into the genome than a DNA-based vaccine. Moreover, mRNA is a safer vector than DNA as mRNA carries a short sequence to be translated, is a transient molecule, and does not interact with the host genome. Third, protein-based vaccines are often produced from bacteria, whereas mRNA vaccines are translated by the host translation machinery thus likely to form an antigen that mimics the protein's structure expressed from the viral genome including the post-translational modifications.

However, the storage and transportation of mRNA vaccines require ultralow temperatures, whereas protein-based vaccines can be stored and transported in less stringent conditions. It has been tested that the leading COVID-19 mRNA vaccines remain stable up to 24 hours at room temperature 37. Thus, it is a huge technical hurdle and economic burden to store and transfer millions of mRNA vaccines to and in warm countries and regions. Nevertheless, with the development of lipid nanoparticle technologies, the stability of mRNA vaccines can be sustained at less stringent conditions 38.

In addition to conventional mRNA vaccines, there is another type of RNA vaccines called self-amplifying RNA (saRNA) vaccines that have been tested and evaluated. saRNA vaccines can replicate after delivery, thus expressing more target antigens in a host at lower doses than conventional mRNA vaccines 39. saRNA vaccines are genetically engineered replicons derived from self-replicating single-stranded RNA viruses and can be delivered as viral replicon particles or as a completely synthetic saRNA produced after IVT. They have been developed and tested in multiple animal models and humans against infectious diseases such as rabies, influenza, RSV, HIV, and Ebola 40.

## mRNA vaccine development for COVID-19

### Optimization of mRNA vaccine design

Typical vaccine development using live-attenuated or inactivated virus or a pseudovirus system involves tedious and time-consuming steps and has become a bottleneck for responding to an epidemic or pandemic caused by newly emerging viruses. As described above, mRNA vaccines possess distinctive advantages of rapid development and versatility as exemplified by the swift development of multiple COVID-19 mRNA vaccines. More importantly, recent preliminary data from clinical trials have shown that two licensed mRNA vaccines, mRNA-1273 and BNT162b, have higher protective efficacy than ChAdOx1 vaccine developed using a chimpanzee adenovirus (~95% vs. ~70%) 4, 41.

However, some intrinsic features of mRNA molecules demand special strategies to guarantee the stability, efficacy and safety of mRNA vaccines. First, mRNA are intrinsically unstable and prone to degradation due to the omnipresence of RNases in the serum and plasma 42. Second, the cellular machinery recognizes exogenous RNA molecules as immunological mimic of viral infection, which results in an immediate immune response 43. Thus, it is a prerequisite for the design of mRNA vaccines to maximize the stability of RNA and translation efficiency and avoid the innate immune response by host cells 44, 45. Below we will discuss the major strategies used in designing mRNA vaccines, including 5'-capping, nucleoside modification, codon optimization, and efficient delivery of mRNA molecules with nanoparticles (Table 2).

### 5'-capping of mRNA vaccines

Endogenous mRNA molecules undergo post-transcriptional modifications, including 5'-capping and polyadenylation, for the stability of mRNA and efficient translation. Naturally, 7-methylguanosine cap (m7G) is added to the first nucleotide of a mRNA chain via 5' to 5' linkage. The 2'-OH of the ribose of the first nucleotide is further methylated to form m7GpppNm, also known as cap1. 5'-capping is critical for protecting mRNA from exonuclease activity, facilitating pre-mRNA splicing, and serving as the binding site for eIF4F, the heterodimeric translation initiation complex 46-49. Recent studies have indicated the 5'-cap structure as a major determinant by which the host can discriminate between self vs. non-self mRNA molecules 50-53. A m7GpppNm cap was added to the 5'-end of the majority of the mRNA vaccines reported thus far during their IVT 54-57.

### Optimization of 5'- and 3'-untranslated regions and the length of polyadenylation tail

Regulatory elements in the 5′-untranslated region (UTR) 58 and the length of 3′-UTR 59 increase protein translation. In addition, the polyadenylation (polyA) tail stabilizes mRNA and increases protein translation. Several recent reports have shown that the length of polyA tail is closely associated with the translation efficiency 60. However, the information on 5'- and 3'-UTRs and the nature of polyA signal sequence remains proprietary and undisclosed for the seven reported mRNA vaccines.

### Nucleoside modification during IVT

Kariko, *et al.*, demonstrated that RNA recognition by Toll-like receptors (TLRs) is suppressed via modification of the nucleosides in mRNA molecules 61, 62. Incorporating m5C, m6A, m5U, s2U, or pseudouridine into mRNA molecules abrogates the immune response by evading the activation of TLR-3, -7, and -8 61. For all the seven reported vaccines, pseudouridine was incorporated into the mRNA vaccines in the place of uridine. In addition, the substitution with pseudouridine, m6A, and s2U in RNA molecules suppresses the degradation of RNA by RNase L 63. Thus, the nucleoside modifications not only enhance the stability of RNA but also reduce the innate immune response.

### Purification of IVT

The contaminating impurities during IVT can massively affect the safety of mRNA vaccines once they are introduced to human cells. Even residual amounts of double-stranded RNA and DNA-RNA hybrid molecules can trigger the innate immune response as they can be recognized by the cellular sensors pattern recognition receptors. Various purification techniques have been used to remove residual impurities from IVT reactions for all the seven mRNA vaccines currently on clinical trials. A previous study indicates that the purification of mRNA reduces the expression of type I interferon and increases the protein translation 64. As summarized in Table 2, various purification techniques such as Oligo dT column, LiCl precipitation, and silicone column have been employed to remove contaminants from *in vitro* synthesized mRNA 45.

### Codon optimization

Several parameters have been considered for the codon optimization, which affects the translation efficiency, protein folding, and mRNA abundance. One example is that the GC content in the sequence. Although GC-rich sequences may be problematic for the secondary structure formation of mRNA, the translation efficiency of a GC-rich sequence can be 100-fold higher than that of a GC-poor sequence 65. The translation elongation rate highly depends on the availability of the cognate tRNA species and the optimization of the codon usage to avoid sequences that match rare tRNA species and incorporate sequences that match more abundant tRNA species 66. Moreover, the codon optimization is essential for the mRNA stability as the codon-dependent translation elongation rate has been implicated as a major determinant of the mRNA stability 67. Mechanistically, reduced translational elongation of mRNA with suboptimal codons results in the recruitment of the DEAD-box RNA helicase, Dhh1p, which triggers mRNA decay 68. Two additional codon optimization methods involve the use of the codons with human bias and the maximum adaptation index 69, 70. Other bioinformatics approaches can be explored to further enhance the stability of mRNA, *e.g.*, via design of the secondary structures and prediction of the expression level based on deep learning 71, 72.

### Designing platform and target immunogen for the seven mRNA candidate vaccines

Each of the seven mRNA candidate vaccines was synthesized *in vitro* from a DNA template encoding either the full-length S protein or RBD of SARS-CoV-2 using bacteriophage T7 RNA polymerase. mRNA-1273, CVnCoV, LUNAR-CoV19, and LNP-nCoVsaRNA mRNA vaccines used the template encoding the full-length S protein with 2P substitutions at K986 and V987 positions to produce the stable pre-fusion form of S protein 73. Pfizer/BioNTech have developed two immunogens, the RBD (BNT162b1) and the full-length S protein (BNT162b2). BNT162b2 has been shown to be safer than BNT162b1, especially in older adults in a preliminary clinical trial, and thus was chosen for a phase 3 clinical trial 74. ARCoV vaccines are based on the RBD of SARS-CoV-2. Whereas the sequences of the 5'- and 3'-UTR of the mRNA templates were not revealed in the literature, the 3'-UTR of BNT162b mRNA vaccine derived empirically by screening naturally occurring 3'-UTRs for the highest RNA stability 75. On the other hand, CVnCoV and LNP-nCoVsaRNA were built on the saRNA platform containing a self-replicating replicon of Trinidad donkey Venezuelan equine encephalitis virus (VEEV). The viral protein-encoding gene of the replicon is replaced with a modified S protein-encoding gene of SARS-CoV-2 with two proline mutations in the S2 subunit, K986P and V987P 54, 55. Consistent with the notion that saRNA vaccines can self-amplify after delivery into host cells, the dose used for vaccination was one to two magnitude lower than conventional mRNA vaccines. As shown in Table 2, the dosage range for CVnCoV and LNP-nCoVsaRNA was 2-12 μg and 0.01-10 μg, respectively. In comparison, the typical dose range for the conventional mRNA vaccines was 30-100 μg.

### Packaging mRNA vaccines with lipid nanoparticle (LNPs)

An early study has shown that the transfection efficiency of naked mRNAs is nearly two orders of magnitude lower than that of mRNA bound to lipofectin formulation 27. The lipofectin-based carriers effectively help mRNA delivery into target cells and protect mRNA from RNase 36, 76. The formulation of liposome-based transfection reagents containing cationic lipids has remarkably been improved in recent years 77. In particular, LNPs, composed of proprietary components including positively charged lipids, cationic polypeptides, polymers, micelles or dendrimers, have been widely used for *in vivo* RNA delivery 78, 79. LNPs encapsulate mRNA and assemble it into the stable lipid bilayers, which are then ingested by cells through a variety of endocytosis pathways. Below is the information for packaging of mRNA vaccines with various LNPs.

1. **mRNA-1273**: It was loaded into two proprietary cationic LNPs, WO2017070626 and WO2018115527. Although the exact formulation is not known, the composition of the LNPs was described as follows, SM-102, polyethylene glycol-2000-dimyristoyl glycerol (PEG2000-DMG), cholesterol, and 1,2-distearoyl-sn-glycero-3-phosphocholine (DSPC) 80.

2. **BNT162b mRNA**: It was encapsulated by patented LNPs with improved efficiency of the mRNA delivery according to its clinical trial report (#NCT04368728) 81, 82. The LNPs are composed of ionizable amino lipid, phospholipid, cholesterol and a PEGylated lipid prepared at a ratio of 50:10:38.5:1.5 mol/mol 82, 83. It is interesting to note that BTN162b and mRNA-1273 vaccines are suggested to be shipped and stored at -80˚C and -20°C, respectively 80, 82.

3. **CVnCoV**: It was formulated with a proprietary LNP, referred to as RNActive® technology platform. The LNP consists of four lipid components: cholesterol, DSPC, PEGylated lipid, and a cationic lipid, however the detailed formulation information was not disclosed. CVnCoV remains stable for at least three months when it is stored at 5℃ as suggested by its manufacturer. Moreover, CVnCoV can be stored at room temperature as a ready-to-use the vaccine for up to 24 hours 84, 85.

4. **ARCoV**: It was encapsulated in LNPs of a proprietary composition using a preformed vesicle method and found thermostable at different temperatures, including 4°C, 25°C, and 37°C for up to one week 86.

5. **ARCT-021**: Currently undergoing phase 1/2 clinical trials, it combines two technologies, i.e., saRNA STARR™ and LUNAR® lipid-mediated delivery method. It was designed to enhance and extend antigen expression, enabling vaccination at lower doses 87. In addition, LUNAR® lipids are pH-sensitive and biodegradable, causing minimal lipid accumulation in cells after multiple dosing 87

6. **LNP-nCoVsaRNA**: Developed by Imperial College London using cationic liposome as the carrier, it has just entered phase 1 clinical trial 55.

4. **ARCoV**: It was encapsulated in LNPs of a proprietary composition using a preformed vesicle method and found thermostable at different temperatures, including 4°C, 25°C, and 37°C for up to one week 86.

5. **ARCT-021**: Currently undergoing phase 1/2 clinical trials, it combines two technologies, i.e., saRNA STARR™ and LUNAR® lipid-mediated delivery method. It was designed to enhance and extend antigen expression, enabling vaccination at lower doses 87. In addition, LUNAR® lipids are pH-sensitive and biodegradable, causing minimal lipid accumulation in cells after multiple dosing 87

6. **LNP-nCoVsaRNA**: Developed by Imperial College London using cationic liposome as the carrier, it has just entered phase 1 clinical trial 55.

### RNA candidate vaccines on clinical trials

As of January 22, 2021, 173 candidate vaccines were on preclinical development and 63 on clinical trials. Seven mRNA candidate vaccines (11% of the 63) have completed the preclinical development or are now on clinical trials, of which mRNA-1273, BNT162b, and CVnCoV are undergoing or have completed phase 3 trials. LNP-encapsulated CoVsaRNA, ARCoV, ChulaCov19, and ARCT-021 mRNA vaccines are currently undergoing phase 1 or 2 trials. All vaccines except Lunar-COVID19 were administered at two doses at day 0 and day 21 or 28, respectively. Self-reported adverse effects including pain, swelling, redness in the local injection site, allergy, paralysis, chills, fever, and headache were observed in recipients of three mRNA vaccines mRNA-1273, BNT162b, and CVnCoV during phase 3 trials. Relevant information on all the seven mRNA vaccine candidates has been summarized in Table 3, including the developers, number of doses, dosage, vaccination method, side effects, and stage of clinical trials.

## Delivery route and working mechanisms of mRNA vaccines

According to published preclinical and clinical data, all the seven LNP-encapsulated mRNA vaccines were administered intramuscularly (IM). IM injection is one of the common methods, and the vaccines are injected into deeper tissues under the dermal and subcutaneous layers 88. Shortly after the injection, the LNP-mRNA cargos enter muscle cells through endocytosis and then the mRNA is translated and the translates form metastable trimeric prefusion S protein. Later, a network of blood vessels adjacent to the muscles can recruit infiltrating antigen-presenting cells (APCs).

**Figure 2:**
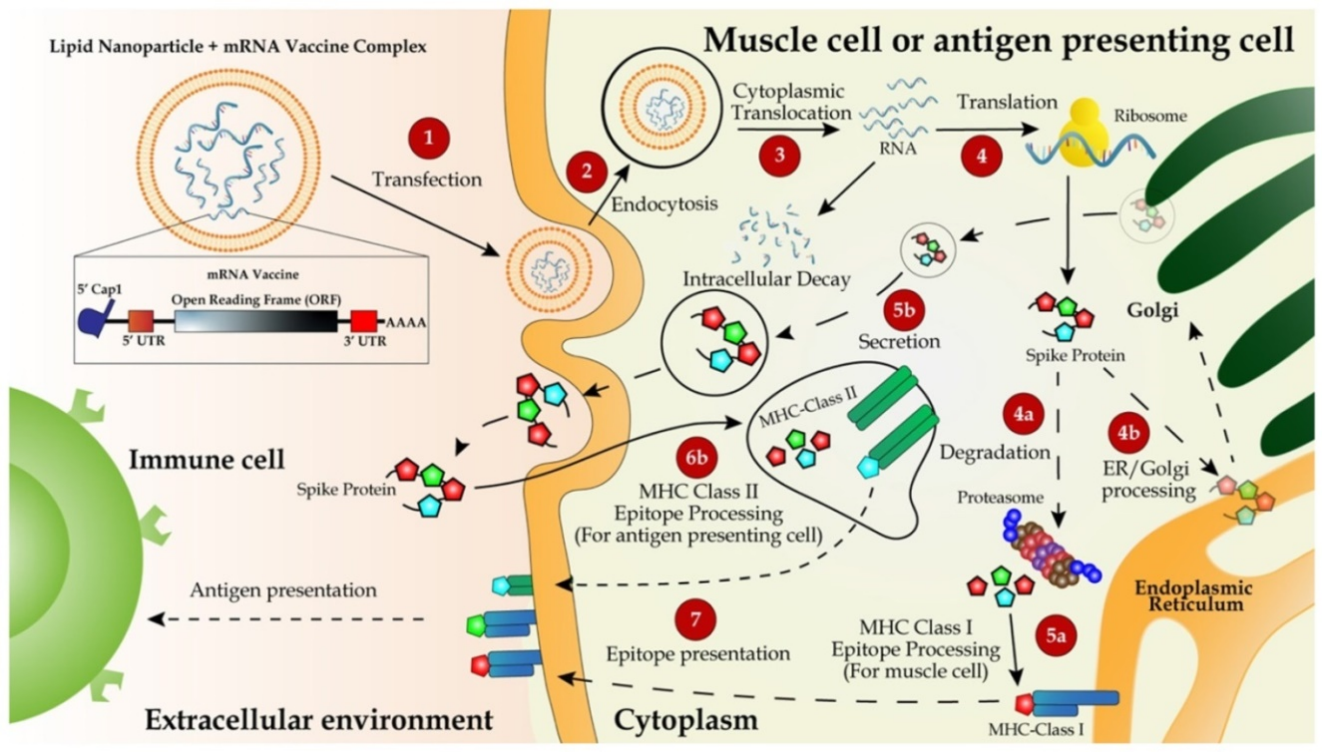
Delivery and working mechanism of a mRNA vaccine. mRNA vaccine, containing the coding region of S protein flanked by the optimized 5'- and 3'-UTRs and polyA tail, is synthesized via IVT, followed by 5'-capping with a 5'-cap analogy and encapsulation with LNP for IM injection (step 1). The vaccine is delivered into muscle cells or antigen-presenting cells such as dendritic cells or macrophages via endocytosis (step 2). mRNA molecules are unloaded from LNPs and translated to S protein in the ribosome (step 3). Newly synthesized S protein is secreted to extracellular space, internalized via endocytosis into antigen-presenting cells and incorporated as a part of MHC class II antigen presentation complex (steps 5b, 6b, and 7) to present the antigen to immune cells including T and B cells 132. Partially degraded S peptides by proteosomes are incorporated into MHC class I complexes, which are then transported to plasma membranes and also presented as antigens to immune cells (steps 4a, 4b, 5a, and 7).

One superior advantage of mRNA vaccines is their use of the cellular translational machinery and other cytosolic components in producing a properly folded and fully functional protein from each injected mRNA. In the case of mRNA vaccines designed with the full-length S protein, the translated product contains a signal peptide from amino acids 1 to 15, enabling the S protein to be transported to the plasma membranes or secreted out of the cytoplasm. Meanwhile, the majority of the protein will be degraded in endosome-derived proteasome and subsequently incorporated as a part of the class I major histocompatibility complexes (MHCs), and presented to CD8+ and CD4+ T cells, respectively 53,59. Dendritic cells transfected by an mRNA vaccine or its endocytosed immunogens process the assembly of the class II MHC complex and present it to immune cells (Figure 2).

However, the major mechanism for immunization with an mRNA vaccine is humoral immune response via the activation of B cells. Once naïve B cells are activated by interacting with the cognate CD4+ T cells and the ligation of CD40, the activated B cells will proliferate and differentiate to either memory B cells or antibody-secreting plasma cells in lymphoid organs. The newly activated B cells with a high and low affinity will differentiate to short-lived plasma cells and quiescent memory B cells, respectively 89, 90. Upon the secondary antigen exposure, the circulating antibodies produced from plasma cells will bind and neutralize the antigen, thus blocking the antigen-carrying virus from infecting it target cells. An insufficient amount of antibodies will activate memory B cells either to trigger secondary immune response 91.

## Cellular fates of mRNA vaccines

### RNA degradation

mRNA vaccines took the vaccine development stage by storm mainly due to their rapid development and versatility of design. However, as described above there are two significant intrinsic limitations of mRNA as a vaccine: 1) the instability of mRNA molecules and 2) the activation of the innate immune response. Although it is generally difficult to estimate the degradation rate of a particular messenger RNA in vivo, studies have estimated that the most endogenous mRNA transcripts are rapidly degraded, usually within 10-15 minutes 92, 93. Two pathways degrade mRNA: 1) 5′ to 3′ exonuclease reaction mediated by Xrn1p after de-capping of 5'-methylguanosin; 2) 3' to 5' digestion mediated by a nuclear multi-protein complex called exosome after the removal of polyA tail, which does not require the removal of the 5'-cap 93, 94. Following injection into muscles, synthetically made mRNA likely undergoes rapid RNA degradation by both extracellular and intracellular RNases. Since the half-life of mRNA in the cytoplasm is directly associated with protein expression, it is critical to maximizing the stability of mRNA. 5'-cap protects mRNA from the action of 5' to 3' Xrn1p-mediated exonuclease and enhances the binding to eukaryotic translation initiation factor 4E (eIF4E) 95. Reciprocally, a recent finding shows that eIF4E promotes the 5'-capping of mRNAs, implicating an intimate crosstalk between 5'-cap and the translation initiation 96.

Chan, *et al.*, demonstrates that the rate of mRNA decay is inversely proportional to the kinetics of translational initiation 93. Thus, one way to increase mRNA stability is to promote the translation efficiency by optimizing the codon usage and UTR sequences, which is implemented for mRNA vaccines. It is worth noting that ARCoV, a recently reported mRNA vaccine by Zhang, *et al.*, can be stored at least one week at room temperature without compromising the stability of the vaccine, mainly due to the proprietary protective nanoparticles against ribonucleases 86. This is an important milestone for mRNA vaccines, given the unstable nature of mRNA molecules.

### Potential activation of immune response by exogenous mRNA and impurities in vaccines

The innate immune system has evolved to defend against viral genomes and replicating intermediates via the potent pathogen-associated molecular patterns (PAMPs). PAMPs sense dsRNA through pattern recognition receptors (PRRs) in most cell types and subsequently activate the expression of pro-inflammatory cytokines and type I interferons (IFNs) 97. Poly (I:C) has been long used as a TLR3 agonist to mimic the viral infection and as an immunostimulatory adjuvant for experimental vaccines 98. Unlike other antigenic features of pathogens, such as flagellin or LPS, mRNAs are common to both host and pathogens and thus it requires the cellular machinery to discriminate the non-self mRNA from the self. Exogenously introduced mRNAs are inherently immunostimulatory 45. Therefore, the principle of distinguishing the non-self mRNA from the self is based on the structural distinction, subcellular localization, and availability of the mRNA. For example, dsRNA, 3'-triphosphate RNA, partially degraded or damaged RNAs, and A to I editing level represent the structural signatures for the non-self mRNA and these features activate the innate immune response via PRRs 99.

**Figure 3:**
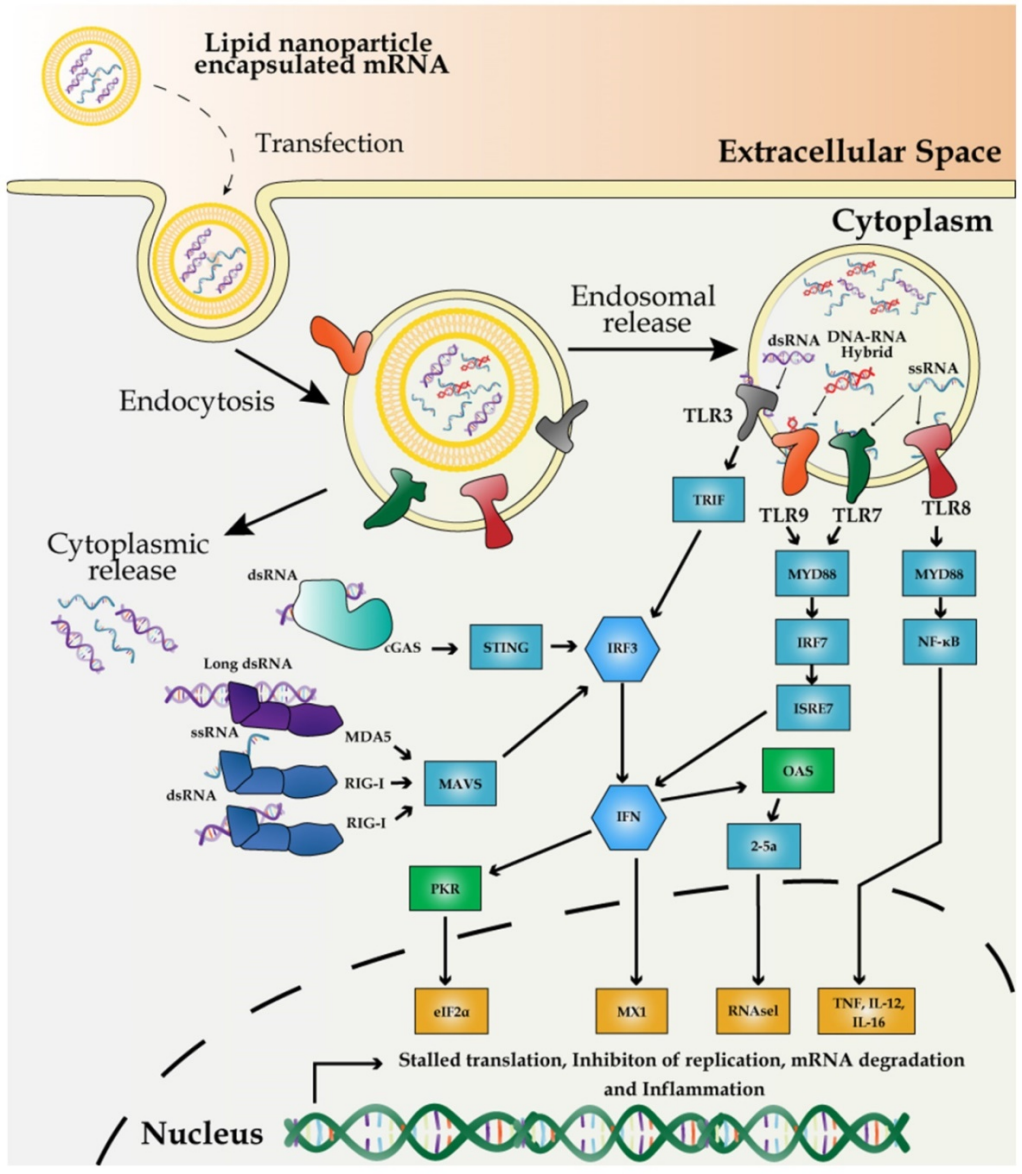
Mechanism of innate immune response to external mRNA. IVT-synthesized mRNA vaccines are recognized by PRRs including the endosomal TLR3, -7, and -8, and cytoplasmic innate immune receptors, RIG-I and MDA5. dsRNA, produced by inaccurate T7 polymerase activity, is recognized by TLR8 and RIG-I to induce the expression of proinflammatory cytokines and promote RNA degradation and translation inhibition mediated by 2′-5′-oligoadenylate synthase/RNase L and PKR-dependent phosphorylation of eIF2α.

RNA impurities during *in vitro* transcription (IVT) of mRNA vaccines potentially trigger the innate immune response, primarily by activating pro-inflammatory genes and type I IFNs (Figure 3). Mainly, dsRNA and DNA-RNA molecules, generated as the by-products of IVT reactions, differentially interact with specific members of PRRs and induce PRR-associated immune responses. Endosome-mediated sensing of long and short dsRNA and ssRNA by TLR family and cytosolic dsRNA sensing via retinoic acid-inducible gene I (RIG-I) are the two pathways most characterized thus far.

First, the primary source of the potentially immune-stimulatory molecule is dsRNA, derived from IVT reaction, in which T7 polymerase transcribes the antisense RNA from the promoter-less DNA template, forming dsRNA via base-pairing with the sense strand 100. dsRNA is recognized by TLR3 in endosomes 101. Alternatively, dsRNA induces antiviral response via another pathway of cGMP-AMP synthase-simulator of interferon genes (cGAS-STING) 102. Second, DNA-RNA hybrid molecules generated during IVT trigger TLR9-mediated sensing of PAMPs and subsequent activation of pro-inflammatory cytokines and type I IFNs in dendritic cells 103. Finally, viral or exogenously introduced single-stranded mRNA (ssRNA) molecules are themselves a PAMP after delivery to host cells, which can also trigger type I IFN production via the endosomal sensors TLR7 and -8 104, 105.

Besides, partially degraded dsRNA in varying sizes can be differentially recognized as dsRNA PAMPs by two cytosolic sensors: melanoma differentiation-associated gene 5 (MDA5) and RIG-I 106. For reducing the potential innate immune response, post-IVT purification has been widely implemented via high-performance liquid chromatography (HPLC) which prevents the activation of type I IFN production 107 and fast protein liquid chromatography (FPLC) which enhances the protein production up to 1,000-fold in primary human dendritic cells 107.

Independent of the innate cellular immune response against the viral and exogenous mRNA 108, dsRNA also triggers the activation of dsRNA-dependent protein kinase PKR, which, in turn, phosphorylates eIF2α, reducing the protein synthesis 109. Thus, the contamination of dsRNA can both trigger type I IFN activation and shut down the protein synthesis. Furthermore, dsRNA activates IFN-induced expression of 2′-5′-oligoadenylate synthetase/RNase L, which promotes RNA degradation 24. In addition, impurities in an mRNA vaccine can also trigger immune response via TLR3 110.

Appropriate purification of IVT-synthesized mRNA is critical to avoid the cellular immune response against the exogenous mRNA and maximize the protein yield. Moreover, the incorporation of chemically modified nucleosides such as pseudouridine and 1-methylpseudouridine allows mRNA molecules to escape the recognition by TLR7 and -8 as well as other innate immune sensors 62, 111. Surprisingly, pseudouridine in mRNA molecules enhances the translation efficiency from ssRNA by reducing the PKR activity 112. Moreover, pseudouridine-modified mRNA can be translated in primary dendritic cells and even in mice by evading innate immune surveillance and increasing the protein yield 62.

## Preclinical and clinical results of COVID-19 mRNA vaccines

Several experimental approaches are often considered to determine the efficacy of any vaccine. Induction of immune response, concentration of antigen-binding IgG, and antigen-neutralizing titres were determined in many preclinical studies for the seven mRNA vaccines. As summarized in Table 4, several mRNA vaccines with their experimental data publicized have demonstrated strong immunogenic activity by inducing both CD4+ and CD8+ T cells. Besides, the dose-dependent geometric mean of the antigen-neutralizing titre was observed on various animal models.

Preliminary results from phase 3 clinical trials have shown that the efficacy of the two mRNA vaccines, mRNA-1273 and BNT162b, reached 95% and 94.1%, respectively, comparably higher than that of another licensed vaccine, ChAdOx1 developed using a chimpanzee adenovirus (Oxford-AstraZeneca) which was 70% based on interim results of a phase 3 clinical trial 4, 5, 41. Both mRNA vaccines were equally effective at all age groups tested for them (Table 5). Among 30,420 volunteers, 15,210 each were assigned in the placebo and vaccine groups in the observer-blinded clinical trial of mRNA-1273. 84.2% (vs. 19.8% in the placebo group) and 88.6% (vs. 18.8% in the placebo group) in the vaccine group reported adverse effects after the first and second doses, respectively. These adverse effects include pain, erythema, swelling, and lymphadenopathy on the injection sites 4. 54.9% and 79.4% of all the participants after the first and second doses, respectively, also reported mild to moderate systemic adverse effects such as fever, headache, fatigue, myalgia, nausea, and chills 4.

As for BNT162b, 43,448 participants were recruited to a placebo-controlled phase 3 clinical trial with 21,728 and 21,720 assigned to the placebo and vaccine groups, respectively. 83% (vs. 14% in the placebo group) and 78% (vs. 12% in the placebo group) of the 16-55 years-old vaccine groups and 71% (vs. 9% in the placebo group) and 66% (vs. 8% in the placebo group) of the 55+ years-old vaccine groups reported mild to moderate local injection-site pain after the first and second doses, respectively. Common local adverse effects include redness and swelling and the systemic adverse effects were mild to moderate including fever, fatigue, headache, chills, vomiting, diarrhea, muscle pain, and join pain 5. Between December 14 and 23, 2020, 1,893,360 doses of BTN162b were administered, of which 1,177,527 doses were for females, 648,327 doses for males, and 67,506 doses missing sex information). 4,393 (0.2%) people reported adverse effects after administration of the first dose of the vaccine, and the adverse effects developed within 30 minutes after the vaccination in 75% of the cases 113. Among 21 people who reported anaphylaxis, 19 (90%) were female and 18 (86%) had allergy history.

Although continuous study of the long-term protection against SARS-CoV-2 and adverse effects remain to be carried out, the efficacy and safety of the two mRNA vaccines appear to be higher than the vaccines against other infectious diseases. According to a meta-analysis of 31 studies associated with the assessed efficacy of the licensed influenza virus, trivalent inactivated vaccine produced 59% efficacy in adults aged 18-65 years 114. Overall, the efficacy of mRNA-1273 and BTN162b was considerably higher than the influenza vaccine against the seasonal influenza.

## Conclusion and perspectives

The COVID-19 pandemic caused by the novel virus SARS-CoV-2 has mobilized a historically great number of scientists, clinicians, and government officials to work together in developing vaccines to cope with the health crisis all over the world. It is remarkable to witness that two mRNA vaccines, mRNA-1273 and BNT162b were developed and manufactured in less than one year. Without the technical advancements in RNA synthesis in a GMP-grade and large-scale manner, nanoparticle formulations, and the “smart” design of RNA vaccines, scientists would not have achieved the feat in producing highly effective and safe RNA vaccines. With the rapidly evolving SARS-CoV-2 and the ever-growing emergence of novel pathogens worldwide, the RNA vaccine platforms will be more widely applicable than ever. A recent screen of the monoclonal antibodies in the blood plasma isolated from mRNA-1273-vaccinated people showed slight to moderate decrease in neutralizing effects against the E484K, N501Y, and the K417N-E484K-N501Y combination variants. However, the rapid design and large-scale production features of mRNA vaccines can reduce the concern that an mRNA vaccine may lose the efficacy against novel SARS-CoV-2 variants.

New and improved methodologies will continue to be explored to optimize the stability and translation efficiency of mRNA and the delivery of LNP-mRNA complexes. Novel approaches, including deep learning and genome-wide screening method to identify the optimal codon usage and UTR design of mRNA are already being tested empirically 72, 115. Recent studies have screened a library of the total mRNA containing 5'-UTR using computational and empirical analyses and determined the optimal 5'-UTR for the maximum RNA stability and translation efficiency in vitro and in vivo 116, 117. Other considerations in codon design, including the GC content, repetitive sequences, secondary structure, and the incorporation of the immunologically less reactive nucleoside analogs, will improve the translation efficiency of mRNA vaccines. It is predictable that saRNA will serve as a more powerful platform than non-replicating mRNA for developing future mRNA vaccines as a smaller dose can produce a sufficient level of mRNA and protein via the self-replicating mechanism. Moreover, direct delivery of mRNA vaccines into antigen-presenting cells such as dendritic cells potentially improves the overall immune response 88.

Regardless of the platform type of the vaccines, there has been a concern about their antibody-dependent enhancement (ADEs). Vaccination often leads to the production of non-effective neutralizing antibodies in the host, which can exacerbate the pathological symptoms by triggering the harmful immunological cascades to facilitate the viral entry and produce excess amounts of cytokines and complements 118. It is difficult to predict ADE of any vaccine based on in vitro antibody-dependent effects or pre-clinical animal studies, due to the incompatibility between human IgG and its counterpart animal receptors 119, 120. Moreover, partially degraded mRNA could be transcribed to truncated proteins and proteins with conformational changes, which then induce the production of neutralizing antibodies that won't bind the native immunogen and can also lead to ADE. Thus, there is a continuous demand to identify and overcome ADE. Nevertheless, after more than a year of massive disturbance and destruction of human lives, mRNA vaccines, together with the other platforms of vaccines, have finally provided a great hope to save mankind from the unprecedented pandemic.

## Figures and Tables

**Figure 1:**
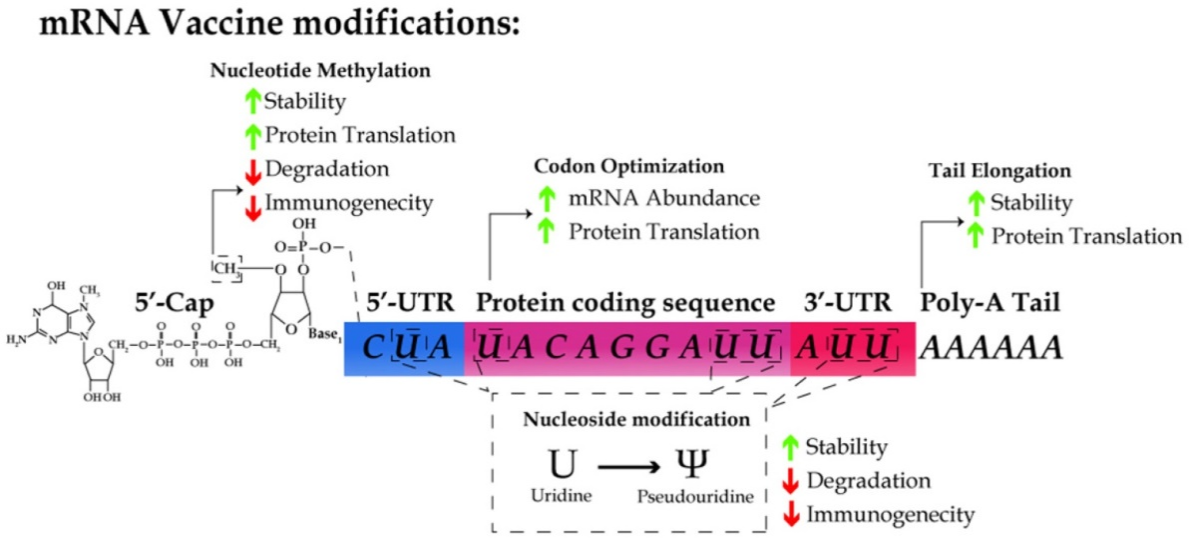
The schematic for designing an mRNA vaccine. mRNA molecules are synthesized *in vitro* with cap1 structure (m7GpppNm), the substitution of uridine with pseudouridine, and the use of the preferred codon in humans, optimized UTR and polyA tail sequence. These modifications results in the increase of RNA stability and translation efficiency as well as the reduction of immunogenicity.

**Table 1:** Current vaccine platforms in clinical trials*

Vaccine platform	Number of candidate vaccines	% of the total number
Protein subunit	19	30%
Viral vector (non-replicating)	10	16%
DNA	8	13%
Inactivated virus	9	14%
RNA	7	11%
Viral vector (replicating)	4	6%
Virus-like particle	2	3%
VVr + antigen-presenting cell	2	3%
Live attenuated virus	1	2%
VVnr + antigen-presenting cell	1	2%

*The data were from the WHO Novel coronavirus Landscape as of January 8, 2021.

**Table 2:** Strategies for developing seven mRNA candidate vaccines

Name of vaccine	IVT pol	5'-cap	Codon optimization	Antigen design	Modified nucleotide	Purification method	Ref.
mRNA-1273	T7	m7GpppNmN	Yes	Full length	N1-methyl pseudouridine	Oligo-dT	56, 121, 122
				S protein			
				K986P/V987P			
BNT162b (3 LNP-mRNAs)	T7	m7GpppNmN	Yes	S protein	N1-methyl pseudouridine	Magnetic purification	
				RBD subunit			57, 123
				K986P/V987P			
CVnCoV	T7	m7GpppNmN	Yes, GC rich	Full length	N1-methyl pseudouridine	LiCl	
				S protein		precipitation	124
				K986P/V987P			
LUNAR-COV19	T7	m7GpppNmN	Yes	VEEV-FL-S	N1-methyl pseudouridine	Silicon column	
				protein			54
LNP-nCoVsaRNA	T7	m7GpppNmN	Unknown	VEEV-FL-S	Unknown	LiCl	
				Protein		precipitation	
				K986P/V987P		LiCl	55
				GGGGSGGGGS linker			
ARCoV	T7	m7GpppNmN	Yes	S protein	Unknown	Unknown	
				RBD subunit			86
				(AA319-541)			
ChulaCov19 mRNA vaccine	Unknown	Unknown	Unknown	Unknown	Unknown	Unknown	NA

Note: IVT, *in vitro* transcription; pol, polymerase; m7GpppN, 7-methylguanosine 5'-triphosphate; VEEV, Venezuelan equine encephalitis virus.

**Table 3:** Seven RNA candidate Vaccines in clinical development as of January 25,221

Vaccine name	# of doses	Dosing schedule	Dosage Tested	Route of administration	Developers	Clinically observevd side effects	Clinical trial phase	Clinical Trial ID	References
mRNA -1273	2	Day 0 + 28	100 ug (Phase 3 result)	IM	Moderna + National Institute of Allergy and Infectious Diseases (NIAID)	Pain, Swelling Redness, Allergy, Paralysis, Chills, Tiredness, Headache	Phase 3	NCT04470427 NCT04283461 NCT04405076 NCT04649151	4, 122, 125, 126
BNT162b2 (3LNP-mRNAs)	2	Day 0 + 21	30 ug (Phase 3 result)	IM	Pfizer/BioNTech + Fosun Pharma	Pain, Swelling, Redness, Allergy, Paralysis, Chills, Tiredness, Headache, Anaphylactic shock	Phase 3	NCT04368728	5, 57, 74, 127, 128
CVnCoV Vaccine	2	Day 0 + 28	2 μg and 12 μg (Phase 1 result)	IM	CureVac/Bayer	Pain, Swelling, Redness, Chills, Tiredness, Headache	Phase 3	NCT04674189 NCT04449276 NCT04515147 NCT04652102 EudraCT-2020-004066-19	124
LUNAR- COV19	1	Day 0	0.2 μg and 10 μg (Preclinical)	IM	Arcturus Therapeutics	N/A	Phase 2	NCT04668339 NCT04480957	54
LNP- nCoVsaRNA	2	ND	0.01 μg to 10 μg (Preclinical)	IM	Imperial College London/ VacEquity Global Health	N/A	Phase 1	ISRCTN170726-92	55
SARS-CoV-2 mRNA vaccine (ARCoV)	2	Day 0 + 14 or Day 0 + 28	100 μg and 1000 μg (Preclinical)	IM	Academy of Military Science (AMS), Walvax Biotechnology and Suzhou	N/A	Phase 1	ChiCTR2000034-112 ChiCTR2000039-212	86
ChulaCov19 mRNA vaccine	2	Day 0 + 21	N/A	IM	Chulalongkorn University	N/A	Phase 1	NCT04566276	N/A

Note: IM, intramuscular; N/A, not apply; ND, not done.

**Table 4:** Preclinical studies on immune response to the seven mRNA candidate vaccines

Vaccine name	Immune reaction	Immune cells	50% inhibitory dilution (ID50) Geometric mean titer		Model	
						Reference
			1st	2nd		
mRNA -1273	Humoral response	Th1 CD4+ T- cells	10 μg: 63 100 μg: 305	10 μg: 103100 μg: 1862	Rhesus Macaque	121
BNT162 (3 LNP-mRNAs)	Humoral and cellular responses	CD4+ T-cells,	Mice:0.2 ug: 26 1ug: 176 5ug: 296	Rhesus macaque:30μg: 962 100μg: 1689	Mice, Rhesus macaque	129
		CD8+ T-cells	Rhesus macaque:30μg: 65 100μg: 81			
CVnCoV	Humoral and cellular responses	CD4+ T-cells, CD8+ T-cells	N/A	N/A	Rhesus macaque	124
LUNAR-COV19	Humoral response	Th1 CD4+ T-cells, CD8+ T-cells	0.2 μg : 57.72 2 μg: 217.9 10 μg: 320	N/A	Mice	54
LNP-nCoVsaRNA	Cellular response	Th1 CD4+ T	N/A	N/A	Mice, Rat	130
ARCoV	N/A	CD4+ T cells, CD8+ T cells	NT50 2ug:278 30ug:559	NT50 2ug: 2540 30ug: 7079	Mice	86
ChulaCov19 mRNA	N/A	N/A	N/A	N/A	Mice, Monkey	131

**Table 5:** Efficacy comparison of approved mRNA vaccines*

	mRNA-1273 Vaccine efficacy % (95% confidence interval)	BNT162b Vaccine efficacy % (95% confidence interval)
Overall	94.1 (89.3-96.8)	95.0 (90.0-97.9)
Age group		
16 to 55 years		95.6 (89.4-98.6)
≥18 to <65 years	95.6 (90.6-97.9)	
> 55 years		93.7 (80.6-98.8)
≥65 years	86.4 (61.4-95.2)	94.7 (66.7-99.9)
≥75 years		100.0 (-13.1-100.0)
Sex		
Male	95.4 (87.4-98.3)	96.4 (88.9-99.3)
Female	93.1 (85.2-96.8)	93.7 (84.7-98.0)

*As of January 26, 2021. Reference: 4, 5

## Abbreviations

COVID-19: Coronavirus disease 2019
SARS-CoV: Severe acute respiratory syndrome coronavirus
MERS: Middle east respiratory syndrome
SARS-CoV-2: Severe acute respiratory syndrome coronavirus 2
ACE2: Angiotensin-converting enzyme 2
IVT: *In vitro* transcription
siRNA: Small interference RNA
saRNA: Self-amplifying RNA
m7G: 7-methylguanosine
eIF4F: Elongation initiation factor 4F
UTR: Untranslated region
TLR: Toll-like receptors
PAMP: Pathogen-associated molecular patterns
RIG-I: Retinoic acid-inducible gene I
RBD: Receptor binding domain
VEEV: Venezuelan equine encephalitis virus
LNP: Lipid nanoparticle
IM: Intramuscular
cGAS-STING: cGMP-AMP synthase-simulator of interferon genes
IFN: Interferon
ADE: Antibody-dependent enhancement
VVr: Viral vector replicating
VVnr: Viral vector non-replicating
